# COVID‐19 Mortality Rates and Changes in Antidementia and Psychotropic Medication Use Among Nursing Home Residents

**DOI:** 10.1002/alz.095099

**Published:** 2025-01-09

**Authors:** Alison C Rataj, Matthew Alcusky, Brian R. Ott, Jonggyu Baek, Shiwei Liang, Kate L Lapane

**Affiliations:** ^1^ University of Massachusetts Chan Medical School, Worcester, MA USA; ^2^ Alpert Medical School Brown University, Providence, RI USA

## Abstract

**Background:**

We examined the relationship between nursing home (NH) COVID‐19 mortality rates and changes in antidementia and psychotropic medication initiation before and during the pandemic and explored the influence of staffing and resident factors.

**Method:**

Changes in medication initiation were collected through a nationally representative cross‐sectional Dementia Treatment Survey (2022) of NH Directors of Nursing. Outcome measures were created by collapsing a 5‐point Likert scale contrasting less/about the same vs. more initiation and less versus more/about the same initiation. NH’s peak monthly COVID‐19 death rate per 1,000 residents (05/2020‐12/2022) was calculated from the CMS COVID‐19 Nursing Home data file. Covariates were drawn from NH Compare, Provider of Service, Master Beneficiary Summary, Minimum Data Set 3.0, and Medicare administrative claims files. Adjusted odds ratios (aORs) were estimated from multivariable logistic models.

**Result:**

NHs with higher COVID‐19 death rates at the peak of the pandemic were more likely to report a decrease (aOR_decrease_: 2.65, 95% CI: 1.07‐6.53) and less likely to report an increase (aOR_increase_: 0.28, 95% CI = 0.09‐0.87) for antidementia medication initiation. No association with psychotropic medication initiation was observed. NH’s reported increased resident behavior problems during the pandemic had higher odds of psychotropic initiation (aOR = 11.25, CI = 3.86‐32.83).

**Conclusion:**

Increased COVID‐19 mortality rates were associated with decreased initiation of antidementia medication use during, compared to before the pandemic. Higher mortality reported with antipsychotic use in this population did not appear to be a factor in NHs during the pandemic. Increased behavior changes may be due to consequences of COVID‐19 (i.e., social isolation); our findings indicate that increased behavior changes were associated with more psychotropic drug use.

